# High-Flow Nasal Oxygen as an Adjunct to Pulmonary Rehabilitation in an Interstitial Lung Disease Predominant Cohort Awaiting Lung Transplantation: Service Description and Preliminary Findings

**DOI:** 10.3390/jcm14217813

**Published:** 2025-11-03

**Authors:** Kathryn Watson, Peta Winship, Caitlin Vicary, Stephanie Stray, Tenae Lurati, Vinicius Cavalheri

**Affiliations:** 1 Physiotherapy Department, Fiona Stanley Hospital, Perth, WA 6150, Australia; kathryn.watson@health.wa.gov.au (K.W.); peta.winship@health.wa.gov.au (P.W.); caitlin.vicary@health.wa.gov.au (C.V.); stephanie.stray@health.wa.gov.au (S.S.); tenae.lurati@health.wa.gov.au (T.L.); 2 National School of Health Sciences, Notre Dame University, Perth, WA 6160, Australia; 3 Curtin School of Allied Health, Faculty of Health Sciences, Curtin University, Perth, WA 6102, Australia; 4 Allied Health, South Metropolitan Health Service, Perth, WA 6150, Australia

**Keywords:** pulmonary rehabilitation, physiotherapy, chronic lung disease, high flow nasal oxygen, lung transplant, exertional desaturation

## Abstract

**Background/Objectives**: At Fiona Stanley Hospital’s pulmonary rehabilitation program, people awaiting lung transplantation (LTx), whose exertional oxygen requirements are unable to be met with traditional oxygen interfaces, utilize high-flow nasal oxygen (HFNO) to exercise. In this paper, we aim to: (i) describe the characteristics of our service and of the people who have utilized HFNO; and (ii) explore differences between those who survived vs. did not survive whilst awaiting LTx. **Methods**: We conducted a description of the service and a retrospective analysis (from January 2021 to April 2024). The service description included: facility, equipment/cost, staffing/patient ratio, exercise program characteristics, and safety. Inclusion criteria for the analysis were: people actively listed for LTx and completion of three or more exercise sessions on HFNO. Data extracted included patient characteristics, comorbidities, 6-min walk distance (6MWD) prior to commencing HFNO, and survival pre-LTx. Differences between those who survived vs. did not survive whilst awaiting LTx were explored. **Results**: Nineteen patients were included (13 males; age 60 ± 12 yr; 18 with interstitial lung disease). The median [IQR] number of exercise sessions on HFNO was 15 [9; 25]. Eight (42%) patients died whilst awaiting LTx. In those who survived, the median time to LTx was 46 [25; 268] days. Compared to those who died, those who underwent LTx had fewer comorbidities (median: 2 [1; 4] vs. 4 [3; 5], *p* = 0.03). They also tended to be younger and have greater absolute 6MWD prior to commencing HFNO (mean difference, 95%CI: age −8.6 yr, −19.3 to 2.1; 6MWD 55 m, −74 to 185). Associations between dyspnea or body mass index with survival were not demonstrated. This analysis is hypothesis-generating rather than inferential, given the limited sample size. **Conclusions**: Our unique service of high-flow nasal oxygen (HFNO) use in patients participating in pulmonary rehabilitation whilst awaiting lung transplantation is described. Preliminary analysis suggests that, in people utilizing HFNO whilst awaiting LTx, those who underwent LTx had fewer comorbidities than those who did not survive the waitlist period. Larger studies are needed to explore further differences between those who survive vs. those who do not survive whilst awaiting LTx.

## 1. Introduction

One of the cornerstones of non-pharmacological management in chronic lung disease is pulmonary rehabilitation, an individualized exercise program which generally consists of a combination of aerobic and strengthening exercises, often with an education component, and improves exercise capacity, dyspnea, and quality of life [1,2]. Whilst pulmonary rehabilitation was initially developed for patients with chronic obstructive pulmonary disease (COPD), there are growing referrals for patients with interstitial lung disease (ILD), particularly for those in the most severely impaired subgroup, idiopathic pulmonary fibrosis (IPF). This increase in referrals is due to novel antifibrotic therapies, nintedanib and pirfenidone, as well as an increasing prevalence of IPF globally [3], resulting in growing numbers of patients with IPF remaining stable enough for pulmonary rehabilitation referral and for consideration for lung transplant. The benefits of participation in a pulmonary rehabilitation program for patients with ILD are comparable throughout the broad spectrum of ILD-related conditions [4], and are equally effective in this cohort as compared to COPD [2,3,4]. Further, targeted therapies to improve dyspnea and functional capacity are important in IPF due to the strong association with quality of life and mortality in this group [5].

Although pulmonary rehabilitation is useful for those with ILD, they often need to be managed differently from the usual pulmonary rehabilitation cohort of COPD and other, more gradually deteriorating lung conditions. Those with ILD often have a high symptom burden, with extreme exercise-induced hypoxia, secondary to ventilation/perfusion mismatch and limitation in the diffusing capacity for carbon monoxide [6,7]. This can progressively impact the patient’s functional ability and quality of life [8] and is often a challenging barrier to providing effective pulmonary rehabilitation [9].

There is growing interest in utilizing high-flow nasal oxygen (HFNO) in place of low-flow oxygen therapy in this group to facilitate a higher intensity exercise program whilst limiting extreme desaturation [10]. This supported exercise helps to maintain or increase the patient’s physical conditioning whilst awaiting LTx. Further, one of the main barriers to patients being placed on the LTx waitlist is their high body mass index (BMI). It is likely that, by supporting a higher intensity program with HFNO, and avoiding unnecessary interval training due to desaturation, those patients with a high BMI are able to reach their weight loss targets (as directed by their medical team).

High-flow nasal oxygen is delivered via a humidified circuit and facilitates titration of the fraction of inspired oxygen (FiO_2_) from 21% to 100% whilst delivering high flows of air up to 70 L [6,9]. It supports clearance of anatomical dead space and improved alveolar recruitment, which results in improved oxygenation, reduced work of breathing, and improved reports of dyspnea [9,11]. It can also provide some positive end-expiratory pressure (PEEP), which prevents the collapse of critical alveolar units [6]. Additionally, the humidification can improve mucosal dryness and patient comfort during exercise, particularly in those on higher levels of oxygen [11]. Through this ventilatory support, patients are then supported to improve their exercise tolerance and increase the intensity of the exercise session [9]. Additionally, HFNO allows patients to achieve a better exercise performance when compared with standard oxygen therapy (SOT), due to alveolar recruitment and the prevention of early deoxygenation at a muscular level [6]. A recent systematic review [12] identified non-significant but clinically important differences favoring HFNO compared with SOT during CPET testing in several domains (MD [95% CI]); SpO2: HFNO: 7.47% [3.96–10.97], SOT: 5.84% [3.78–7.91], exercise duration HFNO: 144.22 s [23.65–264.79], SOT: 117.77 s [25.14–210.41]; dyspnea HFNO −0.96 [−1.61 to −0.32], SOT −0.27 [−1.05 to −0.26]; and fatigue (SMD [95% CI]): HFNO −0.45 [−0.89 to −0.01], SOT −0.27 [−0.49 to −0.04].

Another potential benefit of HFNO is that it can act as a pulmonary vasodilator and reduce right ventricular afterload in patients with pulmonary hypertension [13], which is a frequent comorbidity in advanced ILD and one that can have a significant impact on the patient’s ability to exercise safely. Additionally, HFNO has been found to be not inferior to non-invasive ventilation (NIV), and is generally more tolerated due to patient–ventilator dyssynchrony, claustrophobia, and mask intolerance associated with NIV [14,15]. Despite this, the efficacy of HFNO use with ILD patients in the critical care setting is controversial, with some indicating that HFNO may simply offer a bridge to escalation of care, rather than have an impact on mortality in acute exacerbations. More research is required to establish the real efficacy of this oxygen delivery method in this setting [16]. Whilst this is not the setting of interest for this paper, it is an important consideration, as many patients awaiting transplant can deteriorate to a level requiring critical care management.

Whilst the benefits of HFNO are well defined from a pathophysiological perspective, and HFNO use is becoming increasingly commonplace in clinical settings for managing patients with advanced lung disease, strong evidence supporting its use in a pulmonary rehabilitation setting is limited. Several studies have demonstrated clinical improvements with the use of HFNO in an exercise setting, particularly in exertional desaturation and physical endurance, but the study populations are highly heterogeneous, with varying degrees of respiratory compromise [10,11,17]. It is not yet clear which respiratory population demonstrates the most benefit from HFNO use, or when the transition should be made from low-flow oxygen options for pulmonary rehabilitation.

In this paper, we will (i) describe the characteristics of our service at Fiona Stanley Hospital (FSH) in Western Australia and of the people who were actively listed, or within 4 weeks of being listed for LTx, who have utilized HFNO; and (ii) explore differences between those who survived vs. those who did not survive whilst awaiting LTx.

## 2. Materials and Methods

### 2.1. Study Design

This is a service description and retrospective analysis using routinely collected data from the pulmonary rehabilitation service within the Advanced Lung Disease Unit at FSH in Western Australia. The retrospective analysis was conducted and reported according to the Strength of Reporting in Observational Studies in Epidemiology (STROBE) Statement and its extension statement, namely REporting of studies Conducted using Observational Routinely-collected health Data (RECORD) [18].

### 2.2. Setting

The pulmonary rehabilitation service within the Advanced Lung Disease Unit at FSH includes metropolitan-based cohorts as well as those from rural outreach locations who are supported by our specialist team. The advanced lung disease team includes: respiratory medical specialists, a clinical nurse consultant, an allied health team of physiotherapists, a social worker, and a clinical psychologist. There are three phases to the pulmonary rehabilitation program, with Phase 1 delivered in the inpatient setting, and Phases 2 and 3 in the outpatient setting for new referrals and maintenance programs, respectively. The current service is described, including information related to: facility, equipment/cost, staffing/patient ratio, exercise program characteristics, and safety.

### 2.3. Study Population, Data Source, Data Collection, and Variables

Clinical outcomes of all patients actively listed, or within 4 weeks of being listed for LTx, who utilized HFNO as part of their pulmonary rehabilitation from January 2021 to April 2024 were included. Data were extracted from the Opal digital medical record (DMR) and i.Clinical manager (iCM).

Inclusion criteria for the outcome analysis were: people actively listed for LTx, and completion of three or more exercise sessions on HFNO either as part of the inpatient or outpatient pulmonary rehabilitation program. This number of sessions was selected to represent at least one week of participation in the intervention being observed. There was no rationale for sample size; rather, the final number of participants is a summary of those who took part in the HFNO program over the timeframe being observed.

Data extracted included patient characteristics such as age, BMI, sex, primary respiratory diagnosis, exertional oxygen prescription (L), number of comorbidities, pre- and post-transplant 6MWT results (6-min walk distance (6MWD), BORG pre and post, SpO_2_ nadir pre and post), transplant outcome, time on transplant waitlist (days), length of ICU and hospital stay (days), number and location of HFNO sessions completed, and transplant-related complications.

### 2.4. Bias

To ensure consistency in data extraction, data were initially extracted by a member of the team (KW) and checked by a second member. All analyses were run by a co-author (VC) who is not a staff member within the pulmonary rehabilitation service in the Advanced Lung Disease Unit.

### 2.5. Statistical Methods

Data analysis was performed using the Statistical Package for Social Sciences (SPSS) version 30.0 (Chicago, IL, USA). To test the distribution of continuous data, the Shapiro–Wilk test and histograms were used. For the description of the characteristics of the participants, categorical variables were reported as percentages, and continuous variables were reported as either mean ± standard deviation (SD) or median [25th; 75th percentile].

Differences between those utilizing HFNO who survived vs. did not survive whilst awaiting LTx were explored via either an independent *t*-test or the Mann–Whitney U test. Variables included in this analysis were: age, BMI, sex, number of comorbidities, 6MWD prior to commencing HFNO, and dyspnea (BORG) at completion of the 6-min walk test. For all analyses, a *p*-value < 0.05 denoted statistical significance.

As this work was registered in the Governance, Evidence, Knowledge, and Outcomes (GEKO) system at Fiona Stanley Hospital and assessed as a Quality Improvement (QI) project, Human Research Ethics Committee (HREC) review and approval were not required.

## 3. Results

### 3.1. HFNO Program

#### 3.1.1. Facility, Equipment, and Cost

The use of HFNO was integrated into the FSH pulmonary rehabilitation program in 2015, when the hospital was commissioned. Both facets of the HFNO program, the inpatient and outpatient services, whilst managed slightly differently, had common goals and targets.

The inpatient service was delivered using the Airvo 2 high-flow system (Fisher & Paykel Healthcare Limited, Auckland, New Zealand), which permits use in rooms that do not have dual medical gas panels, such as the inpatient therapy gym. Typically, patients started at 80% FiO_2_ and were titrated up to a maximum of 100% FiO_2_, depending on the presence of exertional desaturation below 84% SpO_2_. The flow rate was set between 30 and 50 L and titrated as indicated for comfort and tolerance.

The physiotherapy outpatient service was delivered in the outpatient pulmonary rehabilitation gym. The gym space was specifically designed to accommodate patients requiring high-flow oxygen, as the clinicians were consulted regarding space requirements prior to construction of the hospital. Thus, the gym had a dual medical gas panel, which facilitated the use of the BIRD-Air Oxygen Blender (CareFusion, Yorba Linda, CA, USA) with an MR850 humidifier (HFNO/blender circuit) (Fisher & Paykel Healthcare Limited, Auckland, New Zealand). As an alternative, the circuit could be used with size CD portable oxygen and medical air bottles, but this had a very limited duration of use before the tanks were depleted. Consumables were of a similar cost to those used with the Airvo 2 circuit. The settings were determined by the patient’s oxygen requirements and tolerance of flow rate. Typically, patients started at 80% FiO_2_ and were titrated up to a maximum of 100% FiO_2,_ depending on exertional desaturation. The flow rate was set between 30 and 50 L and adjusted for comfort and tolerance.

Where patients were experiencing advanced respiratory failure, the HFNO setup may not have been adequate in maintaining the target peripheral oxygen saturation (SpO_2_) during exercise, even if set to 100% FiO_2_. In this instance, 15 L of oxygen via a non-rebreather mask was applied over the top of the HFNO circuit. This maximized FiO_2_ delivery whilst also taking nose and mouth breathing into account. This cohort was generally admitted and managed on the inpatient ward due to the acuity of their condition.

The exercise equipment available in the 180 m^2^ gym space was expansive, with four treadmills, two upright and two recumbent exercise bikes, a Pilates reformer, two leg presses, two arm ergometers, a multi-station weights area, and a free weights area. There was capacity for two HFNO circuits to be wall-mounted concurrently, whilst providing each person with access to a treadmill, bike, and arm ergometer, without disrupting the circuit or each other.

Some patients requiring the HFNO program were located in a rural setting. This cohort could either be managed locally with support or would have to travel to FSH. This depended on whether the satellite service had the equipment, staffing, funding, or expertise to manage a complex HFNO service. Patients who required HFNO could remain at home if their home service could support them, and if they lived within 2 to 3 hours of the tertiary site. Generally, due to the large size of Western Australia, rural patients would relocate to the metropolitan area to facilitate lung transplant listing.

#### 3.1.2. Inclusion Criteria/Patient Selection

The indications for patient selection were as follows: (i) must be on the active lung transplant waitlist or within 4 weeks of being listed; (ii) oxygen requirements were increasing (≥12 L/min); and (iii) had been given medical approval to commence HFNO therapy from the Advanced Lung Disease Unit medical team. The use of HFNO was usually initiated in the outpatient setting. However, if the patient deteriorated beyond the point of being able to manage at home, their care may have been transferred to an inpatient admission whilst awaiting LTx. These inclusion criteria have remained stable over many years. However, patient numbers have fluctuated depending on the medical criteria for acceptance for LTx listing. Worldwide, LTx eligibility varies depending on local factors including—but not limited to—organ availability and medical expertise.

#### 3.1.3. Staffing

The service in both the inpatient and outpatient settings was staffed and supervised by a Senior Physiotherapist with a 1:1 patient ratio in the first instance. Other outpatient respiratory services were run concurrently, and there were always two dedicated senior staff members in the area while HFNO sessions were running, with clinical experience ranging from 16 to 30 years.

#### 3.1.4. Program Description

As with all pulmonary rehabilitation programs, the HFNO program was tailored specifically to meet patient needs and took any patient limitations into account. There were components of aerobic and strengthening exercises, and these often included elements of interval training to achieve greater work rates, adequate rest, and recovery of SpO_2_ levels. The primary goal of HFNO was to facilitate exercise at a higher intensity to target specific pre-transplant goals. This may have included pre-transplant weight loss targets or maintaining or increasing exercise capacity and strength with the aim of improving post-operative outcomes. Exercises were individually prescribed and included: treadmill, recumbent exercise bike, leg press, step-ups, sit-to-stands, upper limb exercises with dumbbells, kettlebells, and arm ergometer. The program focused primarily on lower limb strength and endurance but incorporated some upper limb strengthening as indicated based on individual goals. Masimo Rad-57 oximeters (Masimo, Irvine, CA, USA) were utilized during cardiovascular training components to monitor SpO_2_ and pulse rate, with either a finger, ear, or forehead probe, allowing close titration of FiO_2_ as required. Additionally, subjective BORG scores (0–10) were used to monitor patients’ dyspnea rating and to adjust training intensity.

The program was continuously monitored, with all patients completing an exercise log each session where they recorded: FiO_2_ and flow delivered and exercises completed, with details regarding the duration, intensity, SpO_2_ nadir, HR peak, and BORG rating for each. This provided a written record of exercise progression and a way to monitor stability or deterioration over time.

#### 3.1.5. Outcome Measures

Primary outcome measures included: 6-min walk distance (6MWD) and BORG subjective dyspnea score to monitor patients’ subjective responses to exercise progression. A 6-min walk test (6MWT) was completed, following European Respiratory Society/American Thoracic Society guidelines [19], upon initial enrolment and reassessed every 8 weeks (or earlier if any change in exercise tolerance or exertional desaturation was noted). This was usually performed on low-flow oxygen (using a non-rebreather mask (NRBM) or oxymizer pendant), which was titrated to have a comparable FiO_2_ to that being provided via the HFNO circuit. If required, a 6MWT could be performed with a portable HFNO/blender with the power off on the humidifier and with portable oxygen and air bottles.

Body mass index (BMI) was closely monitored, as a secondary measurement, to ensure patients remained within recommended guidelines as set by the medical team—not too high, which could influence fitness and recovery from transplant surgery, and not too low to indicate frailty. The BMI has been found to be an independent predictor of mortality following LTx at both 90 days and 1 year, with increased odds of mortality at a BMI under 20 kg/m^2^ and above 28 kg/m^2^ [20].

#### 3.1.6. Frequency

Inpatients participated in pulmonary rehabilitation from three to five times a week, depending on inpatient caseload requirements. Outpatients attended pulmonary rehabilitation from two to three times a week, class capacity permitting. Both inpatients and outpatients completed 60 min sessions.

#### 3.1.7. Safety

This program followed hospital medical emergency policies and procedures and had a medical emergency trolley located nearby with all staff suitably trained. Patient’s subjective responses, SpO_2_, pulse rate, and blood pressure were closely monitored and rest enforced as indicated. There were no adverse events related to the HFNO program.

### 3.2. Summary of Physiotherapy Led HFNO Service at Fiona Stanley Hospital During Pulmonary Rehabilitation Between 2021 and 2024

Between January 2021 and April 2024, 19 patients utilized HFNO during their pulmonary rehabilitation program (see Table 1 for patient characteristics). They continued this modality until they either received a lung transplant or died while awaiting transplant.

#### Comparison Between Those Who Survived vs. Those Who Did Not Survive Whilst Awaiting LTx

Of the 19 patients, 11 (58%) received a successful LTx and 8 (42%) died either prior to transplant or in the ICU. The comparison between those who survived vs. did not survive whilst awaiting LTx is presented in Table 2. Those who had a successful transplant had fewer comorbidities (median: 2 [1; 4] vs. 4 [3; 5], *p* = 0.03) than those who died. Comorbidities were explored to look for trends; 4 (50%) of those who died had a history of obesity, whilst only two (18%) of those who survived reported the same. Those who had a successful transplant also tended to be younger (mean difference (MD), 95% CI: −9 yr, −19 to 2) and tended to have greater 6MWD prior to commencing HFNO (MD, 95% CI: 55 m, −74 to 185).

## 4. Discussion

This paper summarizes the HFNO program within the pulmonary rehabilitation service at FSH. The experiences and outcomes of 19 patients were explored. Of the 19 patients, 11 (58%) received a successful LTx and 8 (42%) died either prior to transplant or in the ICU. Those who had a successful transplant had fewer comorbidities (median: 2 [1; 4] vs. 4 [3; 5] vs. p = 0.03) than those who died. Although this work includes a small population, with limited data analysis, it is able to demonstrate that patients with advanced lung disease, with extreme exertional desaturation, can be supported to participate in a pulmonary rehabilitation program whilst awaiting lung transplantation with the support of HFNO.

Most patients had a background of ILD (*n* = 17), with the majority having the subgroup of IPF (*n* = 10). Interestingly, none of the patients had a diagnosis of cystic fibrosis, COPD, alpha-1 antitrypsin deficiency, or pulmonary arterial hypertension, which supports the suggestion that the ILD cohort may have more extreme exertional desaturation and potentially benefit more from pre-transplant HFNO. The cohort spent a median of 46 [25; 268] days on the lung transplant waitlist, with a wide variability from 20 days to 818 days. Patients were transitioned from a low-flow oxygen to an HFNO physiotherapy service once their exertional oxygen needs increased to an average of 12.5 L/min and completed 15 HFNO sessions [9; 25], in both inpatient and outpatient capacity.

Whilst this work had limited comparative data, the outcomes can be contextualized against the results of other trials investigating the impact of HFNO during exercise in an ILD population. However, the work in this cohort is limited, with highly heterogeneous populations and severity of disease. The work by Chihara et al. [17] is relevant to that of the current study and looked at participants with chronic respiratory failure, on long-term oxygen therapy (LTOT), in an inpatient pulmonary rehabilitation setting over a 4-week timeframe. They found a significant improvement in 6MWD of 55.2 m ± 69.6 (*p* = 0.04) in the HFNO cohort compared to nasal cannula. However, only 47% of the population had IPF, with COPD and bronchiectasis making up the greater proportion of the group. Harada et al. [10] compared exercise testing on HFNO with a venturi mask (VM) in participants with IPF, and identified improved exercise tolerance, SpO2, and leg fatigue in the HFNO group. However, this study excluded those on long-term oxygen therapy (LTOT), and their cohort had VC, FEV1, and D_LCO_ values above 65% predicted, suggesting a comparatively mild disease compared with the cohort of the current study. Another study by Suzuki et al. [11] did not identify any differences between the HFNO and VM groups for endurance, SpO2, HR, and dyspnea, but had significant variability in participants’ responses to therapy, which was attributed to variations in ILD diagnoses included. Early work in this area is promising, but further research investigating the use of HFNO in more advanced lung disease is warranted.

In a critical care setting, the value of HFNO for improving outcomes for patients with ILD remains unclear due to limited evidence. HFNO likely has a role in the management of acute hypoxemic respiratory failure, but there is limited data in homogenous ILD populations. Evidence for mortality, transplant-free survival benefit, and clinically important endpoints such as intubation rates, ICU length of stay, and transplant bridging outcomes remains limited. Whilst out of scope for the current paper, it should be an area of focus for future prospective research. When exploring the differences between those who survived and those who did not survive, the group who survived had fewer comorbidities and tended to be younger and have a greater 6MWD. When exploring comorbidities, the only one that differed between groups was a previous diagnosis of obesity. Although this difference was detected, this may not be a significant finding, considering patients are required to reach a target BMI prior to being considered for lung transplant listing. Therefore, a patient classified as currently obese would not participate in HFNO sessions or undergo a transplant. There were no other obvious differences between groups, particularly regarding cardiac and neurological comorbidities, or issues around mental health, which may have impacted positive post-transplant outcomes. All patients being considered for lung transplants at FSH must adhere to strict medically directed criteria, receive a very thorough medical workup, and be reviewed by multiple members of the multidisciplinary team, including physiotherapy, social work, clinical psychology, and dietetics. This would explain why the differences between groups are not significant from a medical comorbidities and mental health perspective, as those with significant concerns would be unlikely to be accepted for a lung transplant.

The findings reported in our analysis should be interpreted with caution. Our sample size is relatively small, which limits the certainty and generalizability of our findings and precludes any firm conclusions from being drawn about the impact of comorbidities on clinical outcomes. A multivariate analysis was not undertaken due to the small sample size, and we acknowledge that confounders could not be included in the analysis, limiting the interpretation of our findings. There is a potential referral and selection bias intrinsic to the setting of a tertiary transplant centre. In addition, the retrospective nature of the analysis means that some variables could not be controlled for. However, despite these limitations, this work represents a novel analysis of a highly specialized service, providing valuable insights that can inform future research and clinical practice.

## 5. Conclusions

Interest in the use of HFNO in an exercise setting is growing, with early work demonstrating improved endurance and exertional desaturation during pulmonary rehabilitation with the use of HFNO in place of SOT. Further prospective clinical trials investigating the efficacy of HFNO use in ILD and advanced lung disease cohorts are warranted, with an aim to determine those who may particularly benefit from HFNO use during exercise. Our unique service of HFNO use in patients participating in pulmonary rehabilitation whilst awaiting LTx is described. Preliminary analyses suggest that, in people utilizing HFNO whilst awaiting LTx, those who underwent LTx had fewer comorbidities than those who did not survive the waitlist period. Patients who underwent LTx also tended to be younger and have better functional exercise capacity; however, these findings need to be confirmed in larger, multicentre prospective trials. 

### Future Service Directions

The HFNO service at FSH continues to develop and expand with ongoing growth in referrals of patients with extreme exercise-induced desaturation, with higher care needs as described in this paper. After the data for this report were compiled, the service received funding from the Heart and Lung Transplant Foundation of WA for the purchase of a novel device: the Airvo 3 (Fisher and Paykel Healthcare, Auckland, NZ). Future work will focus on the impact of such novel devices in this complex cohort.

## Figures and Tables

**Table 1:** Characteristics of the 19 patients.

Characteristic	Value
Age, yr	60 ± 12
BMI, kg/m^2^	26.5 ± 4.2
Sex, F (%)	6 (32)
Primary respiratory diagnosis, n (%) Non-cystic fibrosis bronchiectasis Interstitial lung disease Connective tissue disease Combined pulmonary fibrosis and emphysema Hypersensitivity pneumonitis Idiopathic pulmonary fibrosis Unclassifiable Pulmonary velo-occlusive lung disease	1 (5) 17 (90) 1 (5) 2 (11) 1 (5) 10 (53) 3 (16) 1 (5)
Exertional oxygen prescription prior to transplant (L)	13 [8; 15]
Number of comorbidities	3 [2; 5]
Pre-transplant 6-min walk test 6-min walk distance, m Nadir SpO_2_, %	314 ± 132 86 ± 6
** Post-transplant 6-min walk test 6-min walk distance, m Nadir SpO_2_, %	407 ± 51 95 ± 3
Outcome, n (%) Died during the waitlist period Received a lung transplant Died in ICU post-transplant Discharged home following lung transplant	7 (37%) 11 (58%) 1 (5%) 10 (53%)
Time on lung transplant waitlist (days)	46 [25; 268]
* Length of stay ICU Hospital	8 ± 5 28 ± 13
Number of HFNO sessions completed	15 [9; 25]
HFNO Setting, n (%) Inpatient Outpatient Both inpatient and outpatient	5 (26) 7 (37) 7 (37)
Complications Cardiac Respiratory Wound healing Neurological/nervous system Nausea Other	28 9 4 4 4 2 5

Data are presented as mean ± standard deviation or median [25th; 75th percentile], unless otherwise stated. Abbreviations: BMI—body mass index; HFNO—high-flow nasal oxygen; ICU—intensive care unit; SpO_2_—peripheral oxygen saturation. * *n* = 12, ** *n* = 11.

**Table 2:** Comparison between those who survived vs. those who did not survive whilst awaiting LTx.

	Survived (*n* = 11)	Died (*n* = 8)	MD [95% CI] or *p*-Value
Age, yr	56 ± 14	65 ± 5	−9 [−19 to 2]
Comorbidities, n	2 [1; 4]	4 [3; 5]	*p* = 0.03 *
6-min walk distance, m	337 ± 130	282 ± 135	55 [−74 to 185]
BMI, kg/m^2^	25.8 (−5)	27.5 (2.46)	*p* = 0.39
Dyspnea post 6MWT (BORG)	4 [3; 5]	4.5 [4; 6.5]	*p* = 0.41

Data are presented as mean ± standard deviation or median [25th; 75th percentile], unless otherwise stated. * *p* < 0.05. Abbreviations: BMI—body mass index; 6MWT—6-min walk test.

## Data Availability

The raw data supporting the conclusions of this article will be made available by the authors on request.

## Abbreviations

The following abbreviations are used in this manuscript:

HFNO	High-flow nasal oxygen;
ILD	Interstitial lung disease;
IPF	Idiopathic pulmonary fibrosis;
LTx	Lung transplant;
6MWD	Six-minute walk distance;
6MWT	Six-minute walk test.
